# Prevalence of gastrointestinal symptoms in the UK adult population and perceived effects of foods

**DOI:** 10.1007/s00394-025-03780-0

**Published:** 2025-08-23

**Authors:** Catriona L. Thomson, Ada L. Garcia, Christine A. Edwards

**Affiliations:** Human Nutrition, School of Medicine, Dentistry and Nursing, College of Medical, Veterinary and Life Sciences, University of Glasgow, Glasgow, UK

**Keywords:** Gastrointestinal symptoms, Dietary fibre, UK adults, Diet

## Abstract

**Purpose:**

Globally adults are not consuming enough fibre. One barrier to higher fibre intake may be the experience or expectation of gastrointestinal (GI) symptoms including flatulence, bloating and abdominal pain. Identifying experiences of GI symptoms and perceptions of the role foods play could inform dietary advice to increase fibre consumption.

**Methods:**

An online questionnaire explored GI symptoms in UK adults and their perceived association with individual foods (vegetables, fruit, fibre-rich products and other commonly consumed foods/drinks).

**Results:**

Of 516 respondents (85% female, median age 38 years), 72.1% reported no existing GI conditions, yet 86% experienced GI symptoms in the past month (flatulence (72%), bloating (64%) and abdominal rumbling (58%)). The most concerning (causing anxiety or worry) symptoms were bloating (18%), heartburn (16%) and abdominal pain (13%). Among those without declared GI conditions, 71% attributed symptoms to foods/drinks, with 42% avoiding specific items, notably dairy (17%) and vegetables (13%). Food avoidance was more common in respondents with IBS (78%, *P* < 0.05). The most common perceptions were that beans (34%) and Brussels sprouts (23%) cause flatulence, fatty and dairy foods cause bloating (17%) and carbonated soft drinks cause belching (17%).

**Conclusions:**

Although flatulence was attributed to some fermentable fibre-rich foods, this symptom did not worry most respondents. The most concerning symptoms (bloating, pain and heartburn) were more commonly associated with other food categories (fatty, dairy and spicy foods). Reassurance that higher fibre intakes are unlikely to cause worrying symptoms could be important in dietary advice to increase consumption.

## Introduction

Dietary fibre intake in the UK, and in many other countries, is well below recommendations in all age groups, despite continued public health efforts to promote the well-established health benefits [1–3]. In the UK, adults are advised to consume at least 30g fibre per day, however, 91% of the population do not meet this recommendation [4, 5]. Barriers associated with increasing fibre consumption are complex and may be linked to both socio-demographic and modifiable factors such as pre-existing knowledge and beliefs. These include hedonic preferences, resistance to behaviour change, perceived cost and preparation barriers, poor knowledge of the beneficial health impacts of fibre and potential links with gastrointestinal (GI) symptoms [6, 7].

Foods containing low molecular weight dietary fibres (e.g. oligosaccharides and inulin), can have an osmotic effect in the small intestine which in larger quantities may cause abdominal discomfort, rapid delivery of undigested intestinal contents to the large bowel and loose stool [8]. Other dietary components that can also exert osmotic effects in the gut include lactose (found in milk and other dairy products) [9] and polyols (found naturally in certain fruits and vegetables but also used in high amounts as sugar-free sweeteners in foods such as confectionery) [10].

Microbial fermentation of fibre with substantial gas production may be the source of symptoms for some people including abdominal discomfort, bloating, distension and flatulence [11]. Fibre-rich foods which may be associated with increased gas production include legumes, pulses, cruciferous vegetables, onions and garlic, however, dairy products and wheat fibre can have a similar effect [8]. Symptoms following increased consumption of poorly digested carbohydrates may also stem from more solid contents in the colon distending the lumen, a feeling to which some individuals may not be accustomed. Colomier et al. explored the prevalence and burden of self-reported meal-related abdominal pain in a large-scale study of healthy participants (n = 54,127) in 26 countries, including the UK, USA and Australia [12]. In this study 33.8% of participants reported experiencing meal-related abdominal pain, with 11% reporting frequent meal-related abdominal pain (defined as more than 50% of total abdominal pain episodes related to meals). Those experiencing frequent meal-related abdominal pain also reported experiencing a lower physical and mental quality of life compared to those with occasional or no meal-related abdominal pain. This study demonstrates the widespread nature of these experiences and highlights the need to explore which meal components may have the greatest impact. Although participants were recruited on the assumption that they were healthy, 25% of those reporting frequent meal related pain met the Rome IV criteria for irritable bowel syndrome (IBS) based on their symptom reports.

The impact of increased intestinal gas or fluid on the experience of GI symptoms may vary widely and depend on individual perceptions and visceral sensitivity [13]. Individuals with IBS report increased symptoms compared to healthy individuals despite the absence of identifiable structural abnormalities or any considerable difference in intestinal gas production [14–20]. Although the aetiology of IBS is not clear, it is thought that increased visceral sensitivity, altered gut motility, impaired gas handling, carbohydrate malabsorption, the gut microbiota, immune system and gut brain axis all contribute to symptom generation [21].

Expectations of ‘normal’ symptoms may also influence individual tolerance levels. Nationally representative surveys suggest that between 38–61% of Western populations report GI symptoms on a regular basis [22–25]. The most reported symptoms in large scale nationally representative studies in American and European adults were flatulence, heartburn/reflux, bloating, abdominal rumbling/borborygmi, constipation and diarrhoea [23, 24]. An individual’s perceptions of dietary causes of GI symptoms may influence future dietary choices, which could be a concern if excluded foods are fibre-rich [26]. Avoidance of foods associated with GI symptoms has been reported in individuals with IBS and is associated with reduced quality of life, reduced energy, protein, carbohydrate [27] and micronutrient intake [28] and altered microbiome composition [29]. Dietary changes or complete avoidance of foods perceived to cause symptoms were reported by 92% of individuals with IBS compared to 46% of healthy individuals in a study carried out in Ireland (n = 135 IBS and n = 111 healthy participants) [30]. The foods most perceived to cause or worsen GI symptoms in healthy individuals and those with IBS were cereal-based foods (e.g. bread, wheat, pasta and breakfast cereal, spicy foods and vegetables). It is important to explore perceptions of foods particularly those beneficial for health including fibre-rich foods, in relation to GI symptoms, in healthy individuals and in those more likely to experience symptoms (e.g. those with IBS). Adherence to dietary fibre intake recommendations is poor in many countries. Better understanding of potential barriers to fibre consumption may help develop strategies to promote sustained increased intakes.

Therefore, the aim of this study was to explore current experiences of GI symptoms in the general UK population, the perceived effect of foods and factors which may influence this.

## Methods

### Study design

This cross-sectional survey of the UK adult population (aged 18y +) was conducted between September 2020 and February 2021. A minimum sample size of 384 was required based on Cochran’s sample size estimation for studies of large populations where variation of an outcome is not clear [31]. The estimated size of the population of interest (UK adult (aged 18 + years) population) is approximately 55,500,000 [32]. The sample size formula for categorical data described by Cochran was deemed most appropriate within the context of this study [33]. The alpha level a priori was set at 0.05, acceptable margin of error at 5% and, although the standard deviation of the outcomes were not known, an estimated variance of 0.5 was used.

Participants were recruited via social media sites including Facebook, Twitter, LinkedIn, Reddit, forums and via word of mouth. Participants accessed the online survey and study information via a web link. After reading the study information, participants could decide not to take part by closing the web page or they could continue, give their consent for the study electronically and complete the survey.

### Questionnaire

The 8-part, 43 question survey was developed using an online tool (JISC Online Surveys, Bristol) following a review of the literature relating to the assessment of GI symptoms in a clinical research context [25, 34–40]. Information regarding participant demographics, digestive symptoms, impact of symptoms and associations between diet and symptoms was collected (Table 1). After piloting with eight individuals, the questionnaire was shortened and more detail added to the participant information sheet regarding the length of the survey and approximate time required to complete it.

**Table 1:** Summary of content of GI symptom survey questionnaire

Section	Content
1. Demographics	Age, sex, education level, length of residence in UK, diet type, GI conditions, medication, smoking, alcohol
2. GI symptoms	Experience of GI symptoms in past 4 weeks, bowel movements, most common and most concerning symptoms
3. Impact of GI symptoms	Diet, lifestyle, body image, sleep, visceral sensitivity index
4. Perceived causes of GI symptoms	Foods/drinks and avoidance
5. Food choices and GI symptoms	Impact of perceived dietary causes of GI symptoms on food/drink choices
6. Diet	Frequency of consumption of fruit, vegetables, wholegrains, beans and pulses, nuts and seeds
7. Physical activity	IPAQ—International physical activity questionnaire^a^
8. Personality	TIPI – Ten item personality index^b^, Obsessive beliefs and behaviours^c^

^a^[41], ^b^[36] ^c^[42]

### GI symptom scoring

Categorical ordinal response data relating to symptom frequency and severity were used to create a numerical score for each symptom and an overall symptom score for each respondent. Each frequency category was assigned a numerical value: Monthly = 1, 2–3 times a month = 2, Weekly = 3, 2–6 times a week = 4, Daily = 5, More than once a day = 6. Each severity category was also assigned a numerical value: Mild = 1, Moderate = 2, Severe = 3. For each symptom report the frequency numerical value was multiplied by the severity numerical value to create a score for each symptom. The total symptom score for each participant was the sum of the individual symptom scores. The maximum possible symptom score was 162 (maximum score for each individual symptom was 18 (6 × 3) and there were nine possible symptoms to report).

### Foods/drinks and GI symptoms

A list of 32 foods and drinks, including some that have previously been reported in the literature as potential causes of GI symptoms, were included in the questionnaire [43–45]. Foods and drinks were chosen to represent foods which may commonly be associated with GI symptoms (e.g. beans), but also foods which may be less commonly associated with symptoms (e.g. low sugar/fat food and sweets—normal or sugar-free). Respondents could also report additional foods that induce symptoms not included in the list. Respondents were asked whether they associate any of the foods or drinks specifically with any of the following symptoms: flatulence, abdominal bloating, rumbling/borborygmi, abdominal pain, constipation, diarrhoea, heartburn, belching or nausea.

### GI medications

Respondents were asked whether they consumed anti-reflux, antacids or heartburn medication (e.g. Gaviscon, Rennie’s, aluminium hydroxide, magnesium carbonate or proton pump inhibitors (omeprazole, lansoprazole etc.)) and, if so, how often they consumed these.

### Data analysis and statistics

All statistical analysis and data handling was conducted using IBM SPSS Statistics version 28.0 and Microsoft Excel version 16.62. Normally distributed numerical data are presented as mean (standard deviation (SD)) and non-normally distributed data as median (interquartile range (IQR)). Categorical data are presented as counts and % of total/group where appropriate. The survey was set up so that a response was mandatory for most questions to allow submission, eliminating the potential for missing data in complete submissions. Non-response to any free text non-mandatory survey questions was not considered missing data, as these items were optional for respondents. As such, the analysis was conducted using available data for each question, without the need for exclusion due to non-response. Binary or categorical data were analysed using chi squared tests. The purpose of this analysis was to test for associations between certain demographic factors (sex, GI medication use) and experience of symptoms and perceptions of the GI impact of foods. Numerical data were analysed by t test and one way ANOVA if data were normally distributed, and Mann Whitney U tests or Kruskal Wallis if data were not normally distributed. This was to assess differences in GI symptom scores between groups. A *P* value  <  0.05 was considered significant. Association of personality traits and symptoms were assessed by correlation of scores.

### Ethics

Ethical approval for the study was granted by the University of Glasgow, College of Medical Veterinary and Life Sciences Ethics Committee (approval number: 200190172 granted on 29.07.20).

## Results

The median age of the 516 respondents was 38 years (IQR 22), 85% were female and 15% male (Table 2). Nearly all respondents stated that they had lived in the UK for more than 5 years (96%). The education level of the respondents was high, with 77% stating they have obtained a university degree (undergraduate programme (37%) or a postgraduate programme (40%)). Most respondents stated they had no conditions affecting bowel/intestinal health (72.1%). However, 100 (19%) respondents self-reported having IBS and 44 (9%) that they have another bowel/intestinal condition (self-reported food intolerance/allergy, *n* = 14; inflammatory bowel disease *n* = 8; acid reflux *n* = 6; coeliac disease *n* = 5; surgical procedure *n* = 4; diverticular disease *n* = 3; other condition *n* = 4). The original intention of this study was not to compare experiences of individuals with no existing GI conditions to those with IBS, however, given the large proportion of individuals with IBS who completed the survey this additional analysis was carried out. The 44 respondents with GI conditions other than IBS were not included in statistical analysis due to the heterogeneity and low number of this group. Only descriptive statistics for these respondents are presented within the results sections referring to all respondents.

**Table 2:** Demographics of respondents to the GI symptom survey

Characteristic	*n*	%
Number of respondents	516	100
*Sex*		
Females	438	84.9
Males	77	14.9
Other	1	0.2
*UK residence*		
Lived in UK for > 5 years	496	96.1
Diet type*		
Typical UK diet	260	50.4
Mixture of typical UK food and that of other cultures	246	47.7
Food traditional to ethnic group	10	1.9
*Education level*		
Schooling completed at 16y	35	6.8
Schooling completed at 17-18y	77	14.9
Undergraduate programme	191	37
Postgraduate programme	207	40.1
*GI health*		
No diagnosis of any conditions affecting bowel/intestinal health	372	72.1
IBS (self-reported)	100	19.4
Other bowel/intestinal conditions (self-reported)	44	8.5
*Fibre-rich food consumption*		
Considers their own diet to be high in fibre	171	33.1
Eats ≥ 5 portions of fruit and vegetables/day	256	49.6
*Smoking and alcohol*		
Cigarette smokers	28	5.4
Alcohol drinkers	403	78.1
Consume < 1–13 units of alcohol/week	367	71.1
Consume ≥ 14 units of alcohol/week	36	7.0

^*^No additional information regarding what constituted each diet type was given

### General experiences of GI symptoms

Eighty six percent of all respondents reported experiencing at least one GI symptom in the previous 4 weeks. For 91% this was their normal experience and 58% felt that symptoms negatively impact their life. Of those who experienced symptoms, 41% experienced at least one symptom daily and 38% experienced at least one symptom more than once a day. Flatulence was the symptom most commonly experienced more than once a day (31%). The most commonly reported symptom was flatulence (72%) followed by bloating (64%), abdominal rumbling (58%) and abdominal pain (57%). The most concerning symptoms for all respondents were bloating (18%), pain (16%) then diarrhoea (13%).

**Fig. 1:**
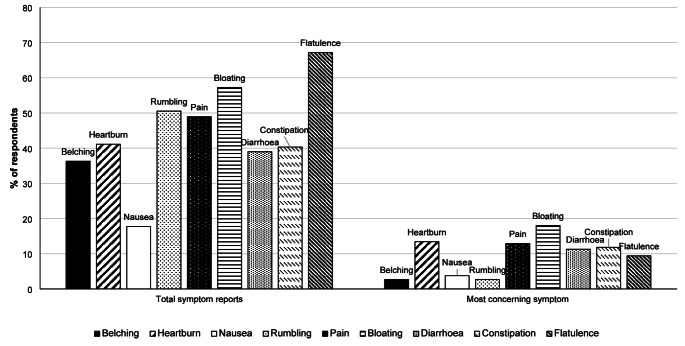
Total symptom reports and most concerning symptom reports for survey respondents with no GI conditions (*n* = 372)

Symptoms were reported by 81% of those with no GI conditions and 99% of those with IBS. Reports of individual symptoms and symptoms which elicited concern in those with no GI conditions are shown in Fig. 1. For those with IBS, flatulence (87%), bloating (85%), abdominal pain (85%) and abdominal rumbling (80%) were the most commonly reported symptoms. For those with IBS the most concerning symptom was pain (26%) followed by bloating (21%), diarrhoea (21%) and flatulence (20%). In relation to symptom scoring, both individual symptom and total symptom scores were generally low. For all respondents, the median total symptom score was 24 (IQR: 34.6) and the highest symptom score was for flatulence (5 (12)) followed by bloating (3 (9.3)) and then rumbling (2 (6)). The highest possible score was 162.

### Food/drinks and GI symptoms

Seventy six percent of respondents reported that specific foods/drinks cause them to experience GI symptoms. Respondents were then asked whether they avoid any foods/drinks that induce symptoms and 52% said that they did. A smaller proportion of those with no GI conditions felt that foods/drinks induce symptoms (no GI conditions: 71%, IBS: 88%) and routinely avoid certain foods/drinks (no GI conditions: 42%, IBS: 78%) compared to those with IBS (*P* < 0.05).

### Perceptions of the contribution of foods and drinks to GI symptoms

**Fig. 2:**
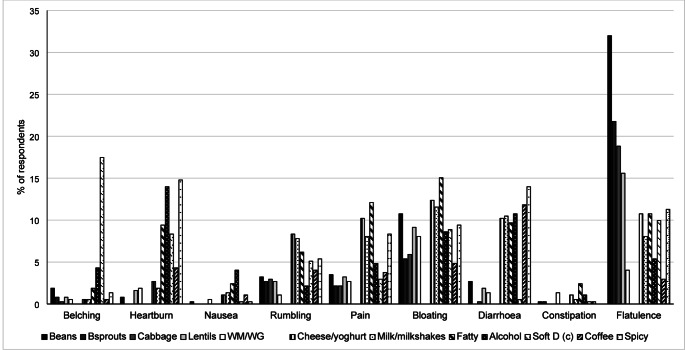
Reports of specific symptoms caused by foods/drinks in survey respondents (N = 372) with no GI conditions. Bsprouts = Brussels sprouts, WM/WG = wholemeal or wholegrain bread, Fatty =—fatty foods, Alcohol = any alcoholic drinks, Soft D (c) = Carbonated soft drinks, Spicy = Any spicy food

Thirty two percent of respondents with no GI conditions reported that beans cause flatulence, 22% felt Brussels sprouts cause flatulence, 17% that carbonated drinks cause belching and 15% and 12% felt that fatty and dairy foods cause bloating respectively. Spicy foods were reported as causing heartburn and diarrhoea in 15% and 14% of respondents respectively and 12% felt that fatty foods cause abdominal pain. The 12 foods/drink categories most commonly associated with digestive symptoms (≥ 10% of total respondents reported) are shown in Fig. 2. A greater proportion of those with IBS reported that beans cause flatulence (41%), spicy foods cause flatulence (25%), fatty foods cause pain (29%) and cheese/yoghurt cause bloating (28%) compared to those with no GI conditions *(P* < 0.05).

### Avoidance of foods

Avoidance of foods/drinks due to perceived symptoms was reported by 52% of respondents. This included 42% of those with no GI conditions and 78% with IBS *(P* < 0.001). For nearly all food/drink categories respondents reported avoiding, a greater proportion of those with IBS avoided these compared to respondents with no GI conditions (Table 3).

**Table 3:** Food categories avoided by respondents with no GI conditions and IBS due to GI symptoms

Food/drink category	No GI conditions *n* (% of respondents)	IBS *n* (% of respondents)
Number of respondents	372 (100)	100 (100)
Dairy products	63 (17)	44 (44)
Vegetables	49 (13)	54 (54)
Drinks	43 (12)	28 (28)
Fizzy drinks	24 (6)	7 (7)
Alcohol	13 (3)	11 (11)
Hot caffeinated drinks	4 (1)	10 (10)
Fruit juice	2 (0.5)	0 (0)
Processed/refined carbohydrates	45 (12)	19 (19)
Spicy foods	23 (6)	13 (13)
High fat foods	16 (4)	5 (5)
Cereals/grains	12 (3)	10 (10)
Fruit	1 (0.3)	13 (13)
Meat	10 (3)	7 (7)
Eggs	5 (1)	7 (7)
Pizza	5 (1)	1 (1)

### Factors influencing symptoms

#### Differences between males and females

Differences in experiences of digestive symptoms or the perceptions of the impact of foods/drinks were identified between males and females For participants without any GI conditions, a significantly greater proportion of females (85.7%) reported symptoms compared to males (61.9%) *(P* < 0.01), stated that foods or drinks induce symptoms (females: 72.7% vs. males: 60.3%) *(P* < 0.05) and stated that they would avoid certain foods or drinks because they induce symptoms (females: 44.8% vs. males: 27.0%) *(P* < 0.05). Women also had higher total symptom scores compared to men (females: median (IQR) 21 (27) vs. males: 6 (19) *(P* < 0.01).

### Personality traits

The TIPI and obsessional behaviour scores of the participants are shown in Table 4.

**Table 4:** Median and Mean TIPI scores and mean obsessional behaviour scores of participants (n = 472)

TIPI scores	Mean	SD
Extraversion	8.0	3.2
Emotional Stability	8.5	2.9
Openness to experiences	9.8	2.2
	Median	IQR
Agreeableness	11.0	3.0
Conscientiousness	11.0	3.0
	Mean	SD
Obsessional behaviour score	27.0	9.3

TIPI scoring [41]: lowest possible score = 2, highest possible score = 14; Obsessional behaviour scoring [42]: lowest possible score = 9, highest possible score = 63

In those with no GI conditions, there was no relationship between the symptom score and extraversion, agreeableness, conscientiousness or openness to experience but there was a negative correlation with emotional stability (r = − 0.221; *p* < 0.01). The symptom score was also weakly positively correlated with the obsessional behaviour score (r = 0.108;*p* < 0.05). There were no correlations between any personality traits and symptom scores in those with IBS.

### Physical activity

Overall, 28% of participants had a high activity IPAQ score, 51% had medium and 21% had low activity scores. There were no significant differences in symptom scores by IPAQ scores in either healthy or IBS groups.

### Medication use and symptoms

Respondents without established GI conditions but who took anti-reflux/antacid medication had a higher symptom score than those who did not *(P* < 0.001) (Table 5). More also reported symptoms when they consume certain foods/drinks and avoid certain foods/drinks because of this *(P* < 0.01).

**Table 5:** Differences between respondents without any GI conditions who take anti-reflux/antacid medication

	Do not take anti-reflux medication (*n *= 273)	Take anti-reflux medication (*n * = 99)	*P*
Total symptom score**^1^	16 (28)	28 (37)	< 0.001
Foods/drinks induce symptoms*^2^	182 (66.7)	81 (81.8)	< 0.01
Avoids foods/drinks because they induce symptoms*^2^	105 (38.5)	51 (51.5)	< 0.01

^*^n (%), ** median (IQR). ^1^Analysed using Mann Whitney U Test, ^2^Analysed using Chi squared Test

A greater proportion of those who take anti-reflux/antacid medication experienced all GI symptoms compared to those who do not take medication, however, this was most notable with the upper GI symptoms belching (no medication: 31.5% vs. anti-reflux medication: 49.5%) and heartburn (no medication: 26.7% vs. anti-reflux medication: 80.8%) *(P* < 0.01).

#### Fruit, vegetable and fibre consumption

Overall, around half of the participants (49.5% no GI conditions; 52% IBS) thought they ate 5 or more portions of fruit and vegetables per day. In both groups, these participants had significantly lower symptom scores than for those who ate less than 5 a day (20.9 vs. 25.8 Healthy; 41.4 vs. 54.1 IBS, *p* < 0.05). There was also a negative correlation between fibre intake and symptoms scores in both groups (r = − 0.143; *p* < 0.05 Healthy; r = − 0.223; *p* < 0.05 IBS).

## Discussion

Reports of GI symptoms were common in this respondent sample, and for many, symptoms negatively impact their lives despite being mild. Although reports of symptoms in our study were higher than has previously been reported, others have found a similar impact of mild symptoms on quality of life in healthy individuals [23–25, 46, 47]. The higher reporting of symptoms in our study compared to past research may be explained in part by the fact our sample was self-selected, therefore, those who chose to participate may have been more aware of their own symptoms than the general population. A higher proportion of our sample reported consuming > 5 portions of fruit and vegetables per day (49.6%) compared to the UK national average (33%) which may also have influenced results [5]. However, this could also be due to differences in cultural norms, customs, beliefs and language between UK adults and other study populations. For example, language differences may mean that interpretation of symptoms differs between countries [48].

Many survey respondents felt that diet was a factor in causing their symptoms (76%) and reported that this may impact their decision to consume certain foods. Previous research into dietary causes of GI symptoms has been predominantly conducted in individuals with IBS, where up to 84% of respondents report symptoms related to foods [44]. Lower GI symptoms were more commonly reported as being caused by foods/drinks in our survey and elicited more concern than upper GI symptoms which is in agreement with previous work in healthy adults [49] and in those with IBS [45, 50, 51]. Foods high in fermentable carbohydrate (beans, Brussels sprouts, cabbage, lentils) were commonly reported as causing flatulence by respondents which was not surprising considering the longstanding perceptions that these foods cause gaseous symptoms [52]. However, those claiming higher fibre and fruit and vegetable intakes in this study had lower symptoms scores than those who ate less. Dairy foods/drinks and fatty foods were more commonly reported as causing abdominal bloating, pain and rumbling compared to plant foods high in fermentable carbohydrates. Reduced activity or expression of lactase may result in the malabsorption of lactose and subsequent digestive symptoms [53]. Lactase activity reduces as we age but varies depending on ethnic group/geographic location [54].

The role of fat in digestive symptoms has been attributed to several mechanisms, which are not fully understood, relating to the sensitisation of gastric perceptions, cholecystokinin release, and intestinal gas accumulation [55]. In individuals who experience bloating following fat consumption, duodenal lipids have been shown to inhibit small bowel motility and impair intestinal gas clearance leading to increased gas retention and bloating [56]. Duodenal lipids have also been shown to increase colorectal sensitivity particularly in individuals with IBS [57].

Spicy foods were also reported to cause several digestive symptoms, diarrhoea, more so than any other food/drink, there were also many reports of heartburn, flatulence and abdominal pain and bloating. The presence of capsaicin in spicy foods can activate TRPV1 receptors in the GI tract causing heartburn and accelerating intestinal transit inducing abdominal pain, loosening bowel movements and increasing the rate of delivery of fermentable substrates to the colon [58, 59].

The belief or expectation that certain foods may induce GI symptoms can influence symptomatic experience [52, 60]. This was demonstrated in a study looking at perceptions of flatulence following bean consumption, where in many cases the anticipation of symptoms was greater than reported symptoms [52]. The potential influence of preconceptions on experience was also observed in a blinded study looking at the impact of a non-absorbable fat substitute (olestra) on GI symptoms [60]. The authors found that after informing all participants of potential GI symptoms they may experience after consuming the fat substitute, very similar proportions of the control (36.9%) and olestra (38.2%) groups reported symptoms. The negative impact of this situation may be the avoidance of foods that may not induce symptoms to the extent individuals perceive. This potentially modifiable barrier to consumption is important to address considering the suboptimal dietary habits of many countries [61, 62].

Although the study was not designed to assess differences between males and females and was therefore not balanced, we found that female respondents were more likely to report symptoms and have negative perceptions of symptoms and food compared to men. This is in agreement with previous findings in irritable bowel syndrome [63–65]. The reasons for this are not clear but could be related to increased symptom reporting in females, increased prevalence of mood or affective disorders and menstrual cycle-related changes in gastrointestinal function and visceral sensitivity [63–65].

We also found that those who take anti-reflux medication experience more symptoms and food-related symptoms compared to those who do not. This study was not designed to assess these differences, however, a considerable proportion of the respondents were taking anti-reflux medication, therefore, it was important to explore the possible impact of this. We did not differentiate between medications so can only speculate as to the potential role of any medication in these symptoms. Perhaps these individuals were more likely to report symptoms and receive medication as a result. It is also well established that some of these medications, proton pump inhibitors particularly, are associated with both short and long term adverse gastrointestinal effects including abdominal pain, flatulence, nausea and diarrhoea [66, 67].

The participants in our study were self-selected, and those who chose to participate may have been more aware of their own symptoms than the general population. However, in general the symptom scores were low for most participants. Another potential limitation is the representativeness of the study respondents. Seventy seven percent of respondents had a university degree (undergraduate: 37%, postgraduate: 40%) which is considerably greater than the UK general population (26–34%) [68, 69]. Also, by advertising and conducting the survey only online, we may have excluded a portion of the population without access to the internet or a device to complete the survey on.

## Conclusion

GI symptoms and perceptions of dietary causes of symptoms were common in this study. Despite flatulence being the most commonly reported symptom, only a very small proportion of respondents were worried by it. Therefore, the perception that foods rich in fermentable fibres (beans, Brussels sprouts and lentils) might cause flatulence, may not be a considerable barrier to increased fibre intake. However, this does demonstrate that some individuals may benefit from reassurance alongside dietary advice that with sustained increased fibre intake symptoms may lessen over time. It is also important to highlight that fibre-rich foods were not most commonly associated with symptoms perceived as being the most concerning (abdominal pain, bloating and heartburn); instead these were associated with fatty, dairy and spicy foods. Creating better public understanding of the relatively low concern expressed of gut symptoms associated with higher fibre foods by individuals and the lower frequency of symptoms in those eating more fibre and fruit and vegetables may help in addressing barriers to increasing fibre consumption.
